# Enzootic Angiostrongyliasis, Guangdong, China, 2008–2009

**DOI:** 10.3201/eid1707.100714

**Published:** 2011-07

**Authors:** Zhen-Yu Qu, Xiao Yang, Mei Cheng, Yan-Feng Lin, Xiao-Ming Liu, Ai He, Zhong-Dao Wu, Xi-Mei Zhan

**Affiliations:** Author affiliations: Sun Yat-sen University, Guangzhou, People’s Republic of China (Z.-Y. Qu, X. Yang, M. Cheng, A. He, Z.D. Wu, X.-M. Zhan);; Qingyuan Center for Disease Control and Prevention, Qingyuan, Guangdong, People’s Republic of China (Y.-F. Lin, X-M Liu)

**Keywords:** angiostrongyliasis, Angiostrongylus cantonensis, rodents, snails, parasites, China, letter

**To the Editor:** The nematode *Angiostrongylus cantonensis* was discovered in pulmonary arteries and hearts of domestic rats in Guangzhou (Canton), China, by Chen in 1935 (*1*). This parasite has a complex life cycle (*2*) and causes cerebral angiostrongyliasis after ingestion of infective larvae found in freshwater and terrestrial snails and slugs, paratenic hosts (such as freshwater fish, shrimp, frogs, and crabs), and contaminated vegetables (*3*).

During 2000–2006, a total of 7 outbreaks of angiostrongyliasis were reported in the People’s Republic of China, including an outbreak in Zhaoqing, Guangdong Province (*4**–**6*). We conducted a survey of *A. cantonensis* nematodes in mollusks and rodents in Qingyuan, Guangong Province, during August 2008–October 2009.

Qingyuan is located in northern Guangdong Province (23°31′–25°12′N, 111°55′–113°55′E). It is the largest city in the province. Qingyuan borders Zhaoqing on the west and Guangzhou on the south. Its climate is subtropical monsoon, and it has an average annual temperature of 20.7°C. The city has an area of 19,152.89 km^2^ and a population of 3.87 million. Nematode hosts were obtained in 3 counties in Qingyuan: Qingxin, Fogang, and Lianzhou.

During August 2008–October 2009, we captured 288 rats of 7 species (257 *Rattus norvegicus*, 13 *R*. *flavipectus*, 7 *R*. *losea*; 6 *R*. *rattus*, 3 *Bandicota* *indica*, 1 *R*. *rattus alexandrinus*, and 1 *Mus musculus*). Rats were examined for adult *A. cantonensis* nematodes in pulmonary arteries and right heart cavities.

Among the 288 rats examined, 27 (9.4%) from 3 species were infected with *A*. *cantonensis* adults in their cardiopulmonary systems (Table). Infected rodents were found in all 3 counties. The 27 infected rats were 25 *R*. *norvegicus*, 1 *R*. *losea*, and 1 *M*. *musculus*. *R*. *norvegicus* rats were most frequently captured in the 3 counties, and this rodent had the highest prevalence of infection. Infected *B*. *indica* rats in Lianzhou and *M*. *musculus* rats in Qingxin were also found, but the total numbers of infected animals and the prevalences are lower than that for *R*. *norvegicus* rats. On the basis of these findings, we conclude that *R*. *norvegicus* rats are the major definitive host for *A*. *cantonensis* nematodes in Qingyuan.

**Table:** Prevalence of infection with *Angiostrongylus cantonensis* in 3 snail species and rodents in 3 counties in Qingyuan, Guangdong Province, China, 2008–2009

County	Host species, no. positive/no. tested (%)			
	*Pomacea canaliculata*	*Achatina* *fulica*	*Bradybaena despecta*	Rodents
Qingxin	12/135 (8.9)	0/32 (0)	3/41 (7.3)	22/137 (16.1)
Fogang	0/9 (0)	6/152 (3.9)	0/19 (0)	2/117 (1.7)
Lianzhou	Not found	0/122 (0)	Not found	3/34 (8.8)
Total	12/144 (8.3)	6/306 (2.0)	3/60 (5.0)	27/288 (9.4)

Specimens from 510 snails (144 *Pomacea canaliculata*, 306 *Achatina fulica*, and 60 *Bradybaena despecta*) were digested with pepsin for isolation of *A*. *cantonensis* larvae (*7*). Metastrongylid larvae were found in 21 (4.1%) of 510 examined snails. Prevalence rates of *A*. *cantonensis* in *P*. *canaliculata*, *A*. *fulica*, *and B*. *despecta* were 8.3%, 2.0% and 5.0%, respectively. Differences between the 3 prevalence rates were significant (χ^2^ 9.604, p<0.05). Prevalence rates in the 3 counties are shown in the Table.

All 3 species of infected snails were found in Qingxin and Fogan Counties. *P*. *canaliculata* and *B*. *despecta* snails were found infected in Qingxin County. However, only *A*. *fulica* snails were found infected in Fogang. These findings are similar to those of studies conducted in Guangdong Province (*8**–**10*).

Distributions of snail species among the 3 sites differed. Although all 3 species were found in Qingxin and Fogang Counties, only *A*. *fulica* snails were found in Lianzhou County. Lower temperatures in this county may contribute to this uneven distribution. Our failure to detect infected snail hosts in Lianzhou County was unexpected, and further surveys are needed to identify parasite hosts in this area. Our findings suggest that the 3 species may play a major role as intermediate hosts for *A*. *cantonensis* nematodes in human infections.

Qingyuan is a natural focus for *A*. *cantonensis* nematodes. Residents in the study area frequently eat raw or undercooked snails and slugs, unaware that these animals may contain infective larvae of *A*. *cantonensis* that can cause eosinophilic meningitis. Therefore, to protect local residents from parasite infections, inhabitants of this region must be given relevant information about *A*. *cantonensis* nematodes. Control measures to control spread of this parasite must also be implemented.
